# Structure and Thermal Stability of wtRop and RM6 Proteins through All-Atom Molecular Dynamics Simulations and Experiments

**DOI:** 10.3390/ijms22115931

**Published:** 2021-05-31

**Authors:** Maria Arnittali, Anastassia N. Rissanou, Maria Amprazi, Michael Kokkinidis, Vagelis Harmandaris

**Affiliations:** 1 Institute of Applied and Computational Mathematics (IACM), Foundation for Research and Technology Hellas (FORTH), IACM/FORTH, GR-71110 Heraklion, Crete, Greece; maria.arnittali@iacm.forth.gr (M.A.); harman@uoc.gr (V.H.); 2 Department of Mathematics and Applied Mathematics, University of Crete, GR-71409 Heraklion, Crete, Greece; 3 Department of Biology, University of Crete, GR-71409 Heraklion, Crete, Greece; amprazi@imbb.forth.gr (M.A.); kokkinid@imbb.forth.gr (M.K.); 4 Institute of Molecular Biology and Biotechnology, Foundation of Research and Technology, GR-70013 Heraklion, Crete, Greece; 5 Computation-Based Science and Technology Research Center, The Cyprus Institute, 2121 Nicosia, Cyprus

**Keywords:** biomolecules, Rop, RM6, proteins, molecular dynamics simulations, mutations, thermostability, secondary structure

## Abstract

In the current work we study, via molecular simulations and experiments, the folding and stability of proteins from the tertiary motif of 4-α-helical bundles, a recurrent motif consisting of four amphipathic α-helices packed in a parallel or antiparallel fashion. The focus is on the role of the loop region in the structure and the properties of the wild-type Rop (wtRop) and RM6 proteins, exploring the key factors which can affect them, through all-atom molecular dynamics (MD) simulations and supporting by experimental findings. A detailed investigation of structural and conformational properties of wtRop and its RM6 loopless mutation is presented, which display different physical characteristics even in their native states. Then, the thermal stability of both proteins is explored showing RM6 as more thermostable than wtRop through all studied measures. Deviations from native structures are detected mostly in tails and loop regions and most flexible residues are indicated. Decrease of hydrogen bonds with the increase of temperature is observed, as well as reduction of hydrophobic contacts in both proteins. Experimental data from circular dichroism spectroscopy (CD), are also presented, highlighting the effect of temperature on the structural integrity of wtRop and RM6. The central goal of this study is to explore on the atomic level how a protein mutation can cause major changes in its physical properties, like its structural stability.

## 1. Introduction

Engineering functional materials at the nanometer scale is a fundamental challenge for nanotechnology [1]. Nature provides peptides and proteins as a major source of inspiration for the engineering of responsive, protein-based nanomaterials for medical and biotechnology applications. Progress in this field however, relies heavily on the development of a detailed and comprehensive understanding of how the amino acid sequences of proteins dictate their structures and physicochemical properties, such as stabilities [2]. These issues constitute the protein folding problem, a still poorly understood puzzle, which lies at the core of all protein engineering projects. Protein folding is linked to a number of different problems including: (i) the thermodynamic balance of intra and intermolecular forces that dictate protein structure for a given amino acid sequence [3]; (ii) the predictability of protein structure from its amino acid sequence, and (iii) the accessible folding pathways that give rise to the observed folding rates of proteins. In view of these complex issues, selected recurrent motifs of protein structure have been frequently used as convenient model systems, which lend themselves, both for understanding aspects of protein folding and stability, and for the development of rational protein design methods for bio-inspired materials. These fields are not yet satisfactorily understood [4,5,6], despite numerous experimental [7,8,9,10,11] and theoretical [12,13,14,15] studies. For example, the thermal stability of natural proteins is particularly challenging, as it is generally limited to a narrow range of temperatures outside of which proteins frequently denature with concomitant loss of function [7]. This temperature range can however be, in some cases, extended through the introduction of mutations or stabilizing agents, making proteins more suitable for biotechnology or biomedical applications. In this context, a serious obstacle in the rational design of more stable proteins is the lack of adequate theoretical methods for the prediction of protein stability, e.g., methods enabling the identification of flexible regions which are frequently associated with lower stability [3]. These regions are attractive protein engineering targets for the introduction of stabilizing mutations. At the level of computational tools, molecular dynamics (MD) simulations constitute a widely used technique for the study of protein structures in aqueous solutions [13,14,16,17,18,19,20,21,22,23,24]. Simulations using all-atom models provide atomistic details in the predicted conformational changes resulting from, e.g., the introduction of mutations or changes in the protein environment (i.e., temperature, pH), etc.

In this work we focus on the folding and stability of proteins from the tertiary motif of 4-α-helical bundles, a recurrent motif consisting of four amphipathic α-helices packed in a parallel or antiparallel fashion [25,26]. Their folding is largely determined by a repeating pattern of hydrophobic and hydrophilic residues, organized on the basis of a seven-residue-repeats (heptads) pattern [27] defined in Figure S1 of the Supporting Material for the two 4-α-helical bundles studied in this work. This simple protein structure motif has been the subject of numerous protein-folding studies and has been exploited as a building block for bio-inspired materials [28,29].

The dimeric RNA-binding ColE1 repressor of primer (Rop) protein is the paradigm of a highly regular 4-α-helical bundle [30,31,32]. Each monomer is an α-helical hairpin consisting of two antiparallel α-helices connected by a short loop. The regular heptad pattern of the Rop sequence is interrupted only in this tight loop region.

Basic structural simplicity makes Rop a very attractive model system for protein folding studies. The loop region has attracted particular attention because it is associated with the discontinuity of the heptads pattern, and is linked with the remarkable ability of several Rop mutants to adopt altered topologies and physicochemical properties including stability [10,18,19,33,34,35]. A striking example of loop variants includes the mutant RM6, in which a continuous pattern of heptad repeats is established through mutagenesis by the deletion of five loop residues. In this “loopless” mutant, the α-helical hairpin structure of the wild-type monomer is converted into a single helix [19,36]. The complete RM6 molecule is a tetramer consisting of four long α-helices, thus being drastically reorganized relative to the dimeric wild-type Rop (wtRop), and thereby becoming a hyper-thermostable protein [36]. RM6 confirms the remarkable plasticity [37] in structure and physicochemical properties inherent to the Rop sequence. So far, the understanding of the complex relationship between heptad periodicity and the structural/physicochemical properties is poorly understood.

To address the above issues, the present study focuses on the role of the loop region in the structure and consequently the differentiation of the physicochemical properties of Rop and RM6, exploring the key factors which can affect them. The thermal stability of both proteins is explored in atomistic detail. Moreover, experimental evidence for the behavior of proteins at higher temperatures is provided. Information deduced from the present work, provides crucial knowledge for the optimization of thermal stability based on short-range changes in protein topologies. In a previous work of ours [13] the stability of the native state of Rop and RM6 proteins at room temperature (300 K) was studied, though atomistic MD simulations, evaluating at the same time the simulation models for both proteins. Here, a detailed analysis is performed, based on key structural parameters, such as α-helix dimensional properties, hydrogen bonding, the Ramachandran plot and pair correlation functions. Structural changes induced by temperature increases and the most sensitive parts of the structure to temperature changes are explored. Experimental data from circular dichroism spectroscopy (CD), are also presented, highlighting the effect of temperature on the structural integrity of wild-type Rop and RM6. The main goal of this study is to explore, on the atomic scale, how a protein mutation can cause major changes in its physical properties.

## 2. Results

### 2.1. Experimental Probing the Thermal Stability of wtRop and RM6

**Figure 1:**
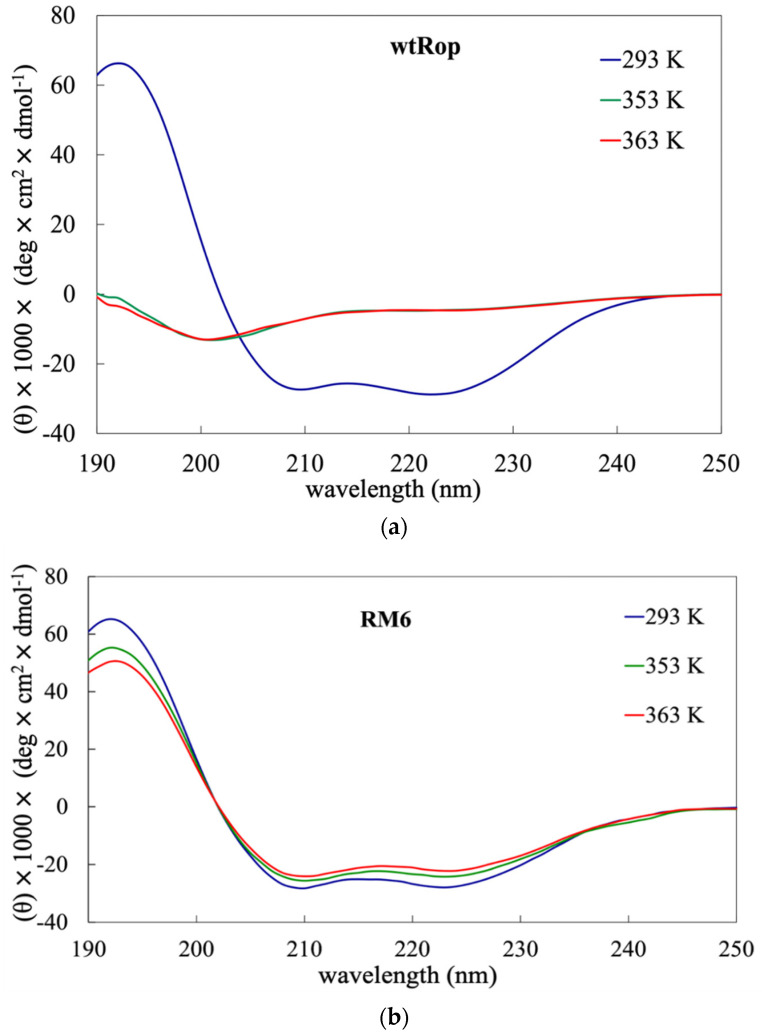
Far UV scans, by CD, for (**a**) wtRop; (**b**) RM6 at three temperatures, 292 K (blue curve), 353 K (green curve), and 363 K (red curve).

Experimentally, the integrity of the secondary structure of wtRop and RM6, has been examined at three temperatures (293 K, 353 K, and 363 Κ) using circular dichroism (CD). At 293 K far-UV CD scans (190–250 nm) exhibited the two characteristic minima (at 208 and 222 nm) of highly α-helical proteins, as the crystal structures indicate (Figure 1). The stability of the proteins reflected in the loss of secondary structure was followed by the loss of the characteristic minima. Experimental data for the far UV scans, for wtRop and RM6 at 353 K and 363 K are presented in Figure 1.

In the case of wtRop, the secondary structure is retained at 293 K but at 353 K and 363 K, it is lost, as the minima of the CD curves indicate. The curves of wtRop, at 353 K and 363 K are characteristic of unfolded protein chains. On the contrary, RM6 is more thermostable and the secondary structure is mostly retained at 353 K and 363 K.

Using the online program BESTSEL [38] for secondary structure analysis of CD data, the α-helical structure of wtRop was verified as being reduced by increasing temperature. At 293 K the structure of wtRop has 73% α-helices but at 353 K and 363 K the percentage is reduced to 10%. This result is consistent with the melting temperature (T_m_) of Rop that is estimated from previous work [37] and is about 333 K. For RM6, a reliable estimate of T_m_ is not possible, since in the accessible temperature range (below 373 K) for the CD system, no noticeable unfolding occurs. As it is shown in Figure 1b, RM6 at 353 K and 363 K still exhibits a considerable α-helical content. The calculated percentage of α-helices is 73% for 293 K and it is slightly reduced to 70% and 68% at 353 K and 363 K, respectively. These data suggest a significantly higher structural stability for RM6 relative to wtRop. All atom MD simulations are consistent with these observations and provide possible interpretations.

### 2.2. Structure Stability of wtRop and RM6 Proteins

In the computational study, we carried out atomistic MD simulations of wtRop and RM6 model proteins at three different temperatures: 300 K, 350 K, and 368 K. Long trajectories have been produced which contain all dynamical information that is needed for our analysis. The model systems that have been simulated in this work are presented in Table 1. Systems involve one wtRop or RM6 protein (Np), and different numbers of solvent (water) molecules (Ns), total number of atoms in the system (N), and ions of Na^+^ (Nions) added to neutralize our systems. All the above, as well as the temperature of the simulation and the sides of the (cubic) simulation box, are shown in Table 1. wtRop protein in systems NSR1, NSR2, and NSR3, and RM6 proteins MRM1, MRM2, and MRM3 are at their native states.

In the upcoming discussion of the simulation results, different names for each subunit of each protein are used. For wtRop protein, the two subunits (monomers) are referred to as ChainA and ChainB. The complete RM6 molecule is a tetramer comprised of four individual subunits (chains A–D). The antiparallel α-helical pair ChainA and ChainB constitutes the asymmetric unit in the RM6 crystals, being symmetrically related to the second α-helical pair (ChainC and ChainD) via a crystallographic (i.e., exact) twofold axis.

#### 2.2.1. Root Mean Square Deviation (*rmsd*)

We start the analysis of the atomistic simulations by examining the stability of both model proteins at their native state. A widely used measure for the calculation of the conformational stability of proteins is the root mean square deviation (*rmsd*) [39,40]. The conformation of the protein is a set of 3D coordinates. We denote the coordinates of the reference structure as $\left\{r_{i}^{ref}\right\}$ (*t* = 0), obtained from the protein data bank, and the coordinates of the protein at any instant time t as $\left\{r_{i}\left(t\right)\right\}$, where $r_{i}=\left(r_{i,x},r_{i,y},r_{i,z}\right)$, i=1,…,N, and N the number of atoms of a protein. In the current analysis, the calculation of *rmsd* was based on the alpha carbon, Ca, atoms, so *N* refers to the number of Ca in the protein. The *rmsd* is calculated as a function of time according to Equation (1), by comparing the equivalent pairs of Ca atoms between the reference and the instant structure:

(1) $$\;rmsd\left(t\right)=\sqrt{\frac{1}{N}\overset{N}{\underset{i=1}{\sum }}{∥r_{i}\left(t\right)-r_{i}^{ref}∥}^{2}}$$

with ∥·∥ being the Euclidean distance between the instant and the reference structure of the *i*th Ca atom.

**Figure 2:**
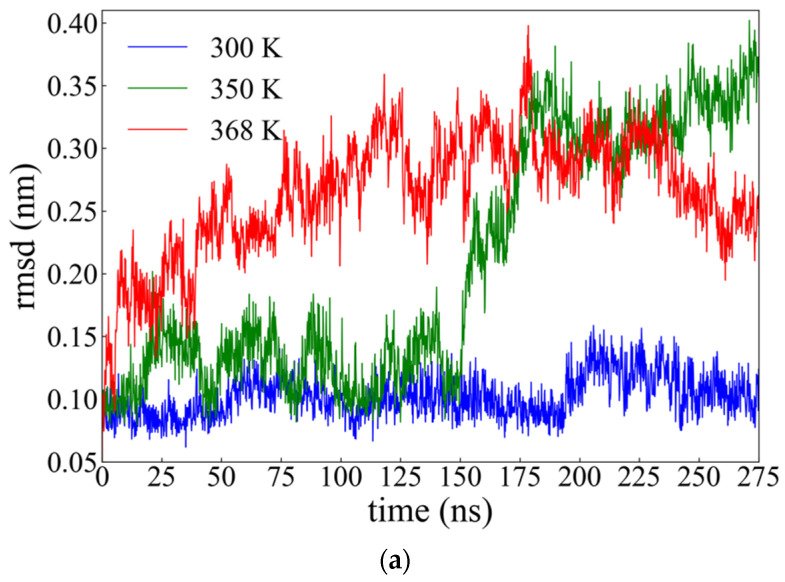

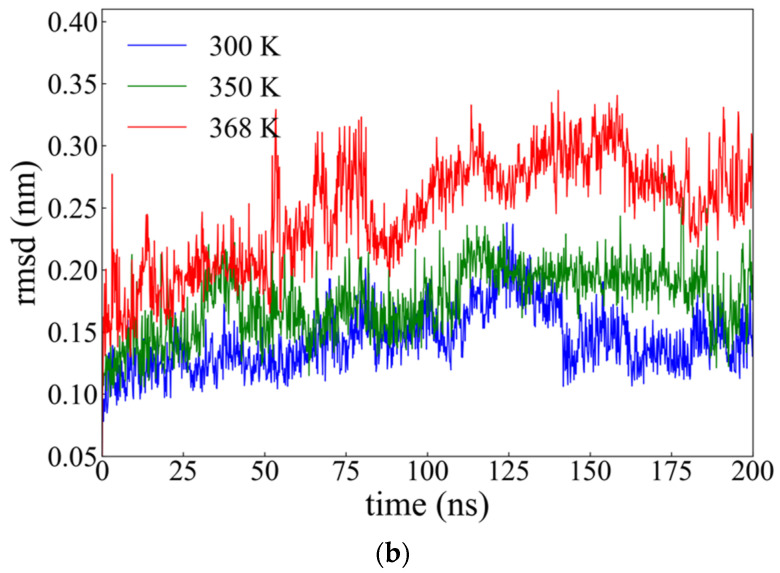
Root mean square deviation (*rmsd*) of Ca atoms, as a function of time of: (**a**) wtRop; and (**b**) RM6; at 300 K (blue), 350 K (green) and 368 K (red).

According to the literature [41,42], *rmsd* values in the range of (0.15–0.25) nm suggest a high degree of similarity to the reference structure. However, the resolution of the experimental structure determination is an important factor for the *rmsd* values; in particular when the initial structure is provided by X-ray crystallography [42], the *rmsd* values tend to increase and their interpretation is harder if the two proteins being compared have been refined crystallographically at different resolutions [42]. In terms of the *rmsd* analysis presented here, the Ca atoms of the seven tail residues of each subunit have been excluded for both proteins, because of the well-known high flexibility of tail parts [43]. Furthermore, in the case of the RM6 protein, the initial four Ca atoms of each chain were also excluded since they were not given in the initial structure. Figure 2a illustrates the *rmsd* values of wtRop as a function of time during the MD simulation at 300 K (blue line), 350 K (green line), and 368 K (red line). All averages and error bars were calculated through average blocking over the last 100 ns of the produced trajectory. At 300 K, it is clear that the *rmsd* is almost stable around ~0.11 nm throughout the whole simulation. This value ensures a rather good simulation model of wtRop [41]. At 350 K, the *rmsd* values seem to be almost stable around the value ~0.14 nm up to ~150 ns whereas, later on an abrupt increase is observed and an almost stable value of ~0.32 nm is attained beyond ~175 ns. So, *rmsd* at 350 K shows a late departure from the structure of the native state, as a result of temperature increasing, attaining a different conformational state. However, with a further increase of temperature to 368 K, conformational change of wtRop is observed immediately and after ~100 ns, the *rmsd* values tend to be stabilized around ~0.30 nm. The bigger values indicate a greater deviation from the initial structure.

The corresponding *rmsd* curves as a function of time of the RM6 protein, at all three different temperatures, are presented in Figure 2b. At both 300 K (blue curve) and 350 K (green curve), the *rmsd* attains similar values, slightly higher at 350 K, (i.e., ~0.15 nm and ~0.19 nm, respectively), remaining almost stable throughout the simulation. At 368 K (red curve), the *rmsd* gradually increases, reaching a value ~0.28 nm, after ~100 ns. Comparing the results for the two proteins, RM6 is found to be less sensitive to temperature increase and hence more thermostable than wtRop in this temperature range.

In Section 2.1, experimental evidence for thermal stability of both proteins in provided. In agreement with the CD results, *rmsd* analysis of our model confirms the instability of wtRop in rising temperatures in contrast to the more stable structure of RM6. However, at the highest temperature value, clear departure from the native state is observed in simulation, whereas a milder change is shown in CD results.

**Figure 3:**
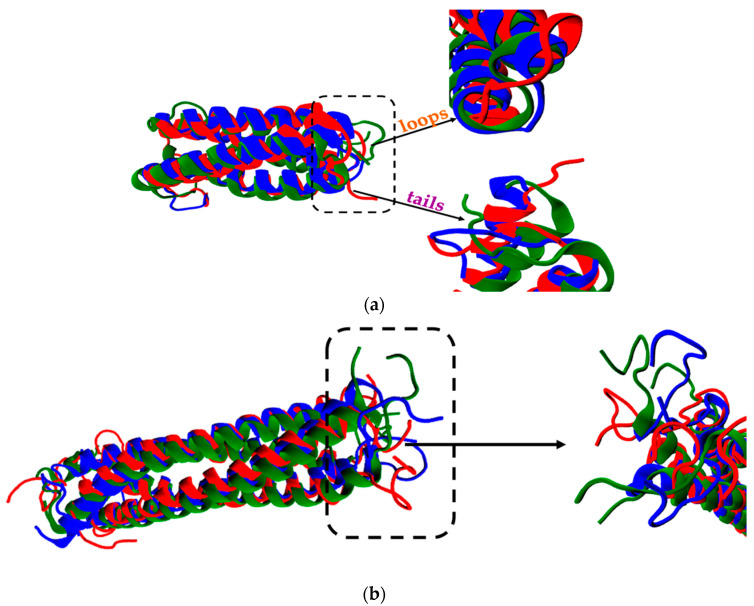
Alignment among the final configurations of (**a**) wtRop; (**b**) RM6 proteins at T = 300 K (blue), at T = 350 K (green), and at T = 368 K (red). Black arrows point to the magnified parts of proteins, which are indicated by dashed frames.

The overlap among the final conformations at the three temperatures (i.e., 300 K (blue), 350 K (green), and 368 K (red)) for wtRop and RM6 is schematically illustrated in Figure 3a,b, respectively, with the use of the VMD tool [44]. A good identification in the conformations for both proteins is observed, in terms of α-helical region. Deviations are obvious in the loop region for wtRop and tail regions for both wtRop and RM6. Concerning the α-helical parts, the overlap at high temperatures is better for RM6.

A further quantification of the thermostability of both proteins is provided by the calculation of the percentage of increase of the *rmsd* values (%*D*), from 300 (which is used as the reference point, since it attains the value of the native state almost constantly) to 350 K and from 300 to 368 K, for both proteins, as a function of time:

(2) $$\%D\left(t_{i}\right)=\frac{\left|rmsd^{T}\left(t_{i}\right)-rmsd^{ref}\left(t_{i}\right)\right|}{rmsd^{ref}\left(t_{i}\right)}$$

**Figure 4:**
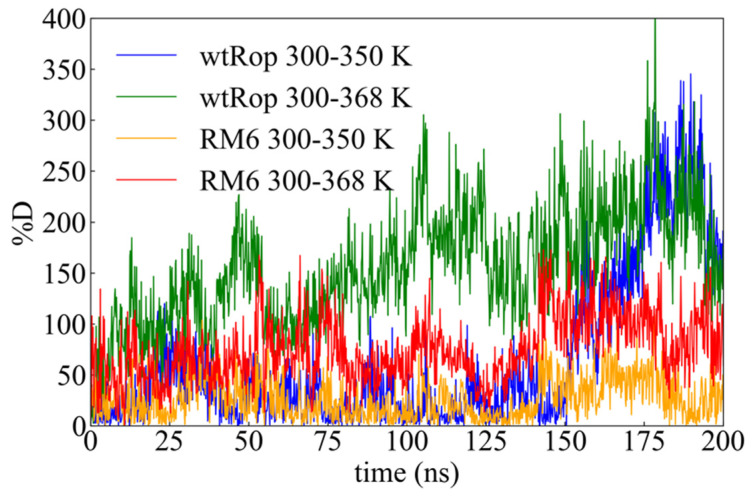
Time evolution of the percentage of increase between the values of the *rmsd* of the reference temperature (300 K) and the two increased temperatures (350 K and 368 K).

In Equation (2), $t_{i}$ refers to instant time, with i=1,…,N and N is the total number of configurations and $rmsd^{ref}\left(t_{i}\right)$ is the *rmsd* value at 300 K, and T stands for 350 K and 368 K. The percentage of increase of *rmsd* as a function of time is shown in Figure 4. The effect of temperature is obviously smaller in RM6, which is rather unaffected up to 350 K, indicating a much higher thermostability in the range of the studied temperatures. Averages over time provide the following values for %*D*: from 300 to 350 K ~224% and ~30% for wtRop and RM6, respectively, and from 300 to 368 K and ~211% and ~89% for wtRop and RM6, correspondingly.

The above findings are in good agreement with experimental data for the melting temperature T_m_ which indicates that T_m_ ≥ 331 K for wtRop, while for RM6, T_m_ ≥ 363 K [37].

#### 2.2.2. Root Mean Square Fluctuation (*rmsf*)

**Figure 5:**
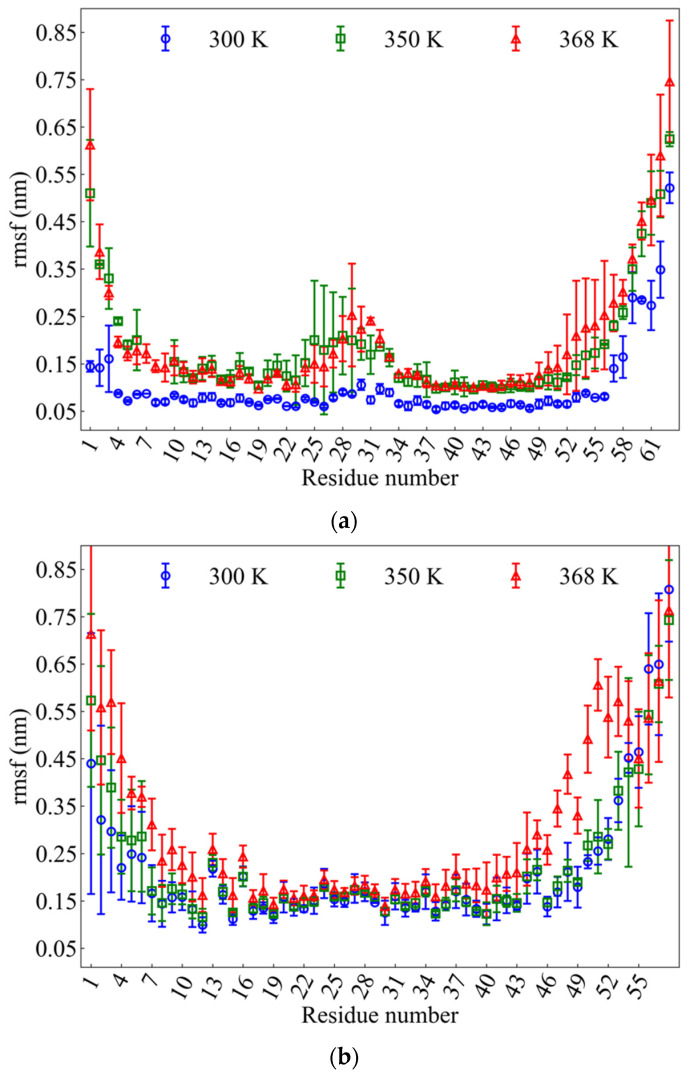
Average root mean square fluctuations (*rmsf*) of Ca atoms between the subunits of: (**a**) wtRop; (**b**) RM6 proteins per residue at 300 K (blue), 350 K (red), and 368 K (green).

In order to gain deeper insight into the most sensitive parts (residues) of proteins to temperature stimuli, we computed the *rmsd* for each individual residue of a protein, which is typically called the root mean square fluctuation (*rmsf*) [22]. The *rmsf* is a numerical calculation for how much a particular residue moves/fluctuates during the simulation. It is plotted versus the residue number and points to the amino acids that contribute the most to the molecular motion. *rmsf* is given by:

(3) $$rmsf_{i}=\sqrt{\frac{1}{T_{R}}\overset{T_{R}}{\underset{j=1}{\sum }}{\left(r_{i}\left(t_{j}\right)-r_{i}^{ref}\right)}^{2}}$$

where *T_R_* is the total time of the simulation, $r_{i}\left(t_{j}\right)$ are the coordinates of atom i of each residue at time $t_{j}$, and $r_{i}^{ref}$ is the reference position of atom i. The computation of *rmsf* was done based on the Ca atom of residue. High *rmsf* values reveal high flexibility whereas low *rmsf* shows limited motion. The time averages of *rmsf* per residue for wtRop and RM6 are shown in Figure 5a,b, respectively. Averaging of *rmsf* values for every residue were also performed on the two chains of wtRop and the four chains of RM6, correspondingly.

Figure 5a illustrates the average *rmsf* values of wtRop protein at three temperatures, versus the residue index. Fluctuations are enhanced at higher temperatures (i.e., 350 K and 368 K) whereas at 300 K motion is limited. Each chain of wtRop protein consists of 63 residues. Special attention has to be paid to the residues which belong to the loop region and their nearest neighbors (residues 25–33), which seem to be more flexible at any temperature. There is an obvious deformation (i.e., jump in *rmsf*) in this region at both 350 K and 368 K which is much less pronounced at 300 K. Different regions of the protein (i.e., N-, C-terminus, α-helices, loop) appear to have different sensitivities at the various temperatures. The higher *rmsf* values correspond to the more flexible parts. Therefore, the most flexible are the residues which belong to N-terminal region (residues 1–3) and to the tail region (residues 57–63), followed by the loop region, whereas the residues of α-helices (3–24 and 34–52) are more stable at any temperature. Moreover, Figure S2 shows the *rmsf* values for each individual subunit of wtRop. Differences in the values of the corresponding residues between the two different chains provide an estimation of the confidential interval for these calculations, which ranges between 0.0–0.10, 0.0–0.12, and 0.0–0.18, for 300 K, 350 K, and 368 K, respectively. The corresponding analysis for RM6 is presented in Figure 5b, where the average *rmsf* values of RM6 protein against the index of the residue are shown. RM6 has 58 residues per chain. The *rmsf* analysis of RM6 mutant reveals higher flexibility of the N-terminal residues as well as the tail residues compared to the α-helices region, similarly to the wtRop, at all three temperatures. Moreover, flexible end regions are more extended in RM6 (i.e., 1–13 and 35–58, respectively), where temperature effect is mostly apparent, whereas the rest part of the chains seems unaffected by the increase of temperature. In agreement with *rmsd* results, this analysis highlights the higher thermostability of RM6 mutant also pointing to the more thermo-sensitive parts of both proteins.

#### 2.2.3. Hydrogen Bonds

At the atomic level, a more detailed investigation can be performed through the computation of the hydrogen bonds (HB) between the various components in all systems, which play an important role in the stability of proteins [45].

In the following analysis, all average values and error bars were calculated through block averaging over the last 100 ns of the trajectory beyond which *rmsd* values remain almost constant. Table 2 contains the average number of HBs between the various components, i.e., protein-protein (P-P), protein-water (P-W), and water-water per water molecule (W-W/W), for all systems studied here. The results reveal a decrease in the number of HBs within the protein molecule by raising the temperature for both proteins (wtRop and RM6). In wtRop there is a reduction of HBs of about 9.7% at 350 K with no further change with temperature rising to 368 K. In RM6 a gradual reduction is observed with temperature increasing which reaches ~8.7% at 368 K. Moreover, the average number of HBs between P and W also decreases when temperature increases. At 368 K this reduction is ~6.43% for wtRop and ~6.8% for RM6. The increased kinetic energy induces conformational changes, as is discussed in detail in a following Section 2.2.5, which are responsible for the reduced hydrogen bonding. The average value of HBs between water molecules per water is comparable to pure water systems, i.e., 3.57 at 300 K [46], 3.361 at 350 K, and 3.277 at 368 K, respectively, according to the specific model. Note here that these numbers attribute hydrogen bonds between waters to both molecules. Very small error bars (less than ~10^−3^) are calculated for the last column data of Table 2 (not included).

A more comprehensive analysis of HB has been performed based on their classification to interchain and intrachain components. Results are presented in Tables S1 and S2, respectively, in the Supporting Material. A decrease of intrachain HBs is observed at higher temperatures, which is attributed to the deformation of α-helices. However, an interesting comparison concerns the hydrogen bonding per amino acid within the chain of each protein (intrachain contribution). More hydrogen bonds are formed in wtRop compared to RM6, which can possibly excuse its smaller helix radius as it will be discussed later. Values of ~0.94 and ~0.83 for wtRop and RM6, respectively, correspond to their native states (i.e., 300 K). Similar differences remain at higher temperatures (i.e., ~0.83 and ~0.77 for wtRop and RM6, respectively, at 350 K; ~0.84 and ~0.72 for wtRop and RM6, respectively, at 368 K).

**Figure 6:**
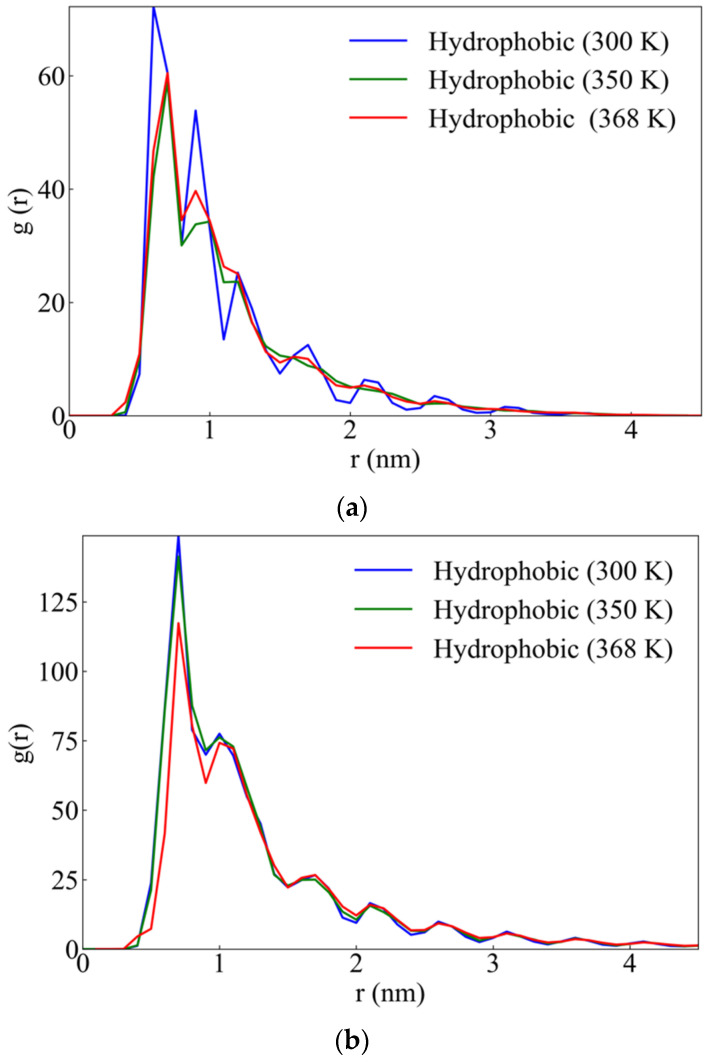
Pair radial distribution function (rdf) between the C_β_ atoms of the interchain/interpair hydrophobic residues at the three different temperatures of: (**a**) wtRop protein and (**b**) RM6 protein.

Using a classification of protein residues analogous to the one used in a recent paper of Kefala et al. [47] (p. 3 Figure 1A), we examined the effect of temperature on the hydrophobic contacts. This calculation provides a manifestation of the way that temperature increasing affects the hydrophobic core, through a possible loss of hydrophobic contacts, or a general rearrangement of all protein residues, which can induce attenuation of the hydrophobicity of the core. The pair radial distribution functions (rdf) between the interchain hydrophobic residues (i.e., ChainA-ChainB for wtRop) and between the interpair hydrophobic residues (i.e., chains A,B-chains C,D for RM6), provide a measure of the proximity between the hydrophobic contacts. rdfs have been calculated between the C_β_ carbon atoms of the hydrophobic residues at various T-values, for both proteins and are presented in Figure 6. A gradual decrease of the first peak with rising temperature is observed, which indicates a reduced probability for their approach, thus a loss of hydrophobic contacts. Moreover, temperature effect is observed from 350 K and beyond for wtRop, whereas for RM6 it appears gradually and becomes more pronounced at 368 K. A similar conclusion is drawn from the calculation of the distance between the centers of mass (CM) of all C_β_ carbon atoms which belong to the hydrophobic residues of each chain/pair for wtRop and RM6, respectively. Hydrophobic residues are mostly oriented towards the interior of the hydrophobic core and their CMs interchain/interpair distance roughly indicates the extension of the core region. Figure S3 in the Supporting Material shows the increases of this distance at higher temperatures, which is more evident for RM6.

##### Alpha Helices

Alpha-helices are defined by a pattern of hydrogen-bonds (HB_α-helix_) between the carbonyl oxygen (C=O) of the *i*th residue and the amide nitrogen (N-H) of the (*i* + 4)th residue (e.g., the C=O of the 3rd residue is hydrogen bonded to the N-H of the 7th residue) (Figure S4) [48]. These HBs are analyzed for all four α-helices of both proteins (each chain of wtRop has two helices connected by a loop). The results are presented in Table 3 where the notation is as follows: Ca and Cb denote the ChainA and the ChainB, respectively, whereas, indices 1 and 2, in the case of wtRop, indicate the two helices of each chain. For RM6, Ca, Cb, Cc, and Cd indicate ChainA, ChainB, ChainC, and ChainD, respectively. A general trend of decrease of the number of HB with the increase of temperature is found, which can be attributed to the extension of the α-helices, however the trend is not systematic with temperature.

##### Hydrogen Bonds of Loop Region

We now turn our attention to the four residues (29Leu (blue), 30Asp (orange), 31Ala (green), and 32Asp (purple) of the loop region, as shown in Figure 10c. According to the references [13,43], among the loop residues, 31Ala is the only one which creates HBs with both α-helices of a chain simultaneously, acting as a bridge between them. Moreover, *rmsf* analysis in Section 2.2.2, shows that residues of the loop are highly flexible at high temperatures. Therefore, the study of the HBs of these loop residues was performed at the various temperature values, in order to examine destruction of hydrogen bond bridges or possible formation of new ones. Results, which are contained in Table 4 for all three temperatures, show that the bridge of 31Ala at 300 K is destroyed at 368 K, whereas a new bridge is formed by 30Asp at 350 K which is destroyed again at 368 K.

#### 2.2.4. Ramachandran Plot

A better insight into the conformational state of proteins is achieved through the Ramachandran plot [49,50,51]. A Ramachandran plot is a phase diagram of two sequential torsion angles, φ, ψ ($\varphi =\left(-C_{i-1}-N_{i}-Ca_{i}-C_{i}\right),$ and ψ $=\left(-N_{i}-Ca_{i}-C_{i}-N_{i+1}\right)$).

**Figure 7:**
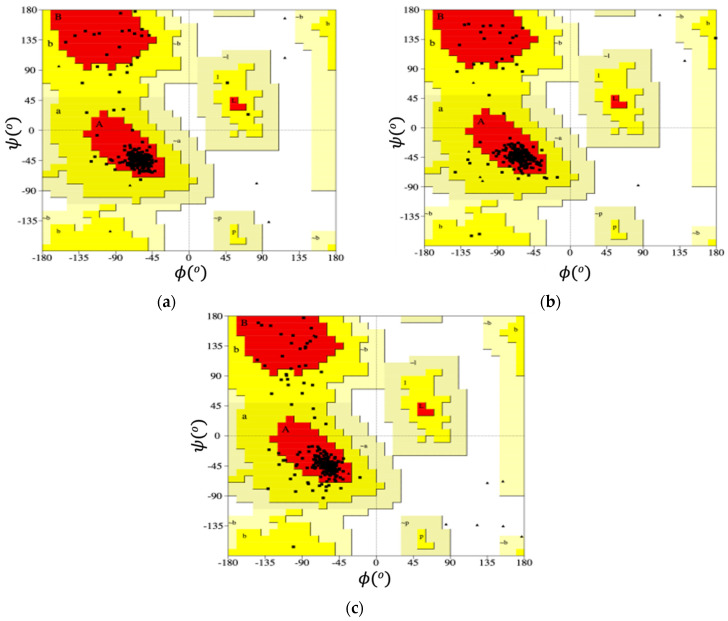
Ramachandran plot of RM6 protein at three different temperatures: (**a**) 300 K, (**b**) 350 K, and (**c**) 368 K. In a typical Ramachandran plot colors indicate how favorable an area is for a combination of (*φ*,ψ) angles. The red shows the most favorable region, whereas the gradient of yellow from darker to brighter regions shows the passage from more to less favorable conformations.

Ramachandran plots of RM6 protein for the combinations of (*φ* − ψ) angles, at the various temperatures, are presented in Figure 7. These are produced using the PROCHECK tool [52,53]. All the residues of wtRop and RM6 are identified by squares with the exception of Gly residue, which is represented by triangles. Each black square represents the conformation of the backbone of every residue of the protein. The different regions of the Ramachandran plots are presented by shading that is obtained from data of high-resolution crystal structures. The darker they are, the more favorable the combination (*φ* − ψ). The most preferred regions are illustrated with red and are marked with A, B, and L, which correspond to the α-helix, the β-strand, and the left-handed α-helix conformations, respectively. The gradient of yellow from darker to lighter regions indicates the passage from more to less favorable conformations. Further details about the different regions of the Ramachandran plot are given in the Supporting Material. We observe that the majority of points are clustered in the area which is represented with red (marked with A) since both proteins attain α-helices secondary structures. Fewer points are present in the red (B) region which corresponds to β-sheets conformations. The corresponding plots for wtRop are shown in Figure S5 in the Supporting Material, with almost identical features. The effect of temperature is captured through this measure as well, by the counting of the percentage of the (*φ*/ψ) angles that exist in the α-helix and in the β-sheet regions, respectively, in all the studied systems. For wtRop, decrease of this percentage from ~86% at 300 K to ~77% at 350 K and ~76% at 368 K in the α-helix region is observed, with a corresponding increase for the β-sheet region from ~6% at 300 K to ~8.0% at higher temperatures. In the case of RM6, a similar decrease from ~85% at 300 K to ~82% at 350 K and ~75% at 368 K in the α-helix region is found, followed by an increase from ~6% to ~9.0% in the β-sheet region.

##### Map of Dihedral Angles of the Loop Residues

**Figure 8:**
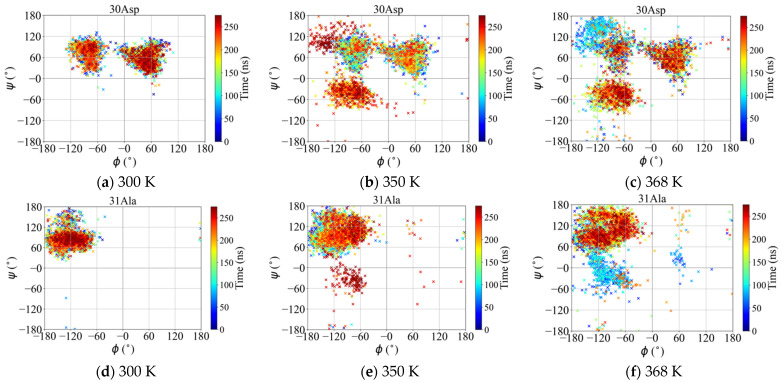
Map plots of all the (*φ*,ψ) torsion angles during the MD simulation. Color code denotes the time instant at which the combination of (*φ*,ψ) is obtained.

Herein, once again the focus is concentrated on the loop residues of the wtRop protein. The time evolution of all the combinations of (*φ*,ψ) dihedral angles during the trajectory for each residue of the loop is recorded and mapped in isosurface plots. The corresponding results for residues, 30Asp and 31Ala, are presented in Figure 8 at all three different temperatures, whereas for the residues, 29Leu and 32Asp, are shown in Figure S6. The color scale bar to the right side of each plot indicates the simulation time in ns. Each symbol in the plot shows a combination of (*φ*/ψ) angles and the color indicates the time instant that the current combination is obtained. More broader distributions in time of (*φ*/ψ) combinations are found at higher temperatures for all four residues. This result is consistent with the creation or destruction of new hydrogen bonds from loop residues (30Asp and 31Ala) as well as with the total conformational changes. In addition to loop residues, a corresponding isosurface plot has been made for a residue that belongs to α-helix region (43Asp) at all three temperatures. Results in Figure S7 indicate completely located distributions, unaffected by temperature.

Moreover, it is expected that the values for *φ*,ψ angles of an ideal α-helix are *φ* = −60° and ψ = −50° [54]. Figure S8 contains the average values of the (*φ*,ψ) dihedral angles for each α-helix of wtRop and RM6 proteins, respectively. Average values were calculated by initially averaging all (*φ*,ψ) pairs along the α-helix at each time frame and then with block time averaging over the last 100 ns of the trajectories. Error bars are the standard deviation among the values of the blocks. For RM6, (*φ*,ψ) values are in the range of −63.4 to −61.3° and −44.51° to −39.64°, respectively, at both 300 K and 350 K, whereas, at the highest temperature of 368 K, big changes in torsional angles (i.e., decrease of *φ* and increase of ψ) indicates deformation of α-helices. Quantification of various measures concerning helix dimensions is presented in the next subsection. (*φ*,ψ) values at 300 K, are in a similar range for wtRop (i.e., −63.05 to −57.32° and −44.4 to −41.94°, respectively) as in RM6. T-rising affects wtRop earlier (i.e., at lower temperatures) compared to the RM6 protein. Thus, a decrease of *φ* and an increase of ψ is observed from 350 K and remains at 368 K in the same intervals. This constitutes an additional evidence for the higher thermostability of the RM6 protein at the examined temperature range. Note that the differences among the corresponding values of (*φ*,ψ) between the two/four chains for wtRop/RM6, respectively, provide an estimation for the statistical uncertainty of these values. Differences are of the order of 3.7–4.9%, 5.6–21.4%, and 3.6–9.1%, for wtRop and 0.9–3.8%, 2.0–2.6%, and 2.4–15.17%, for RM6, at 300 K, 350 K, and 368 K, respectively.

#### 2.2.5. Local Conformation Analysis of Alpha-Helices: Helix Properties

**Figure 9:**
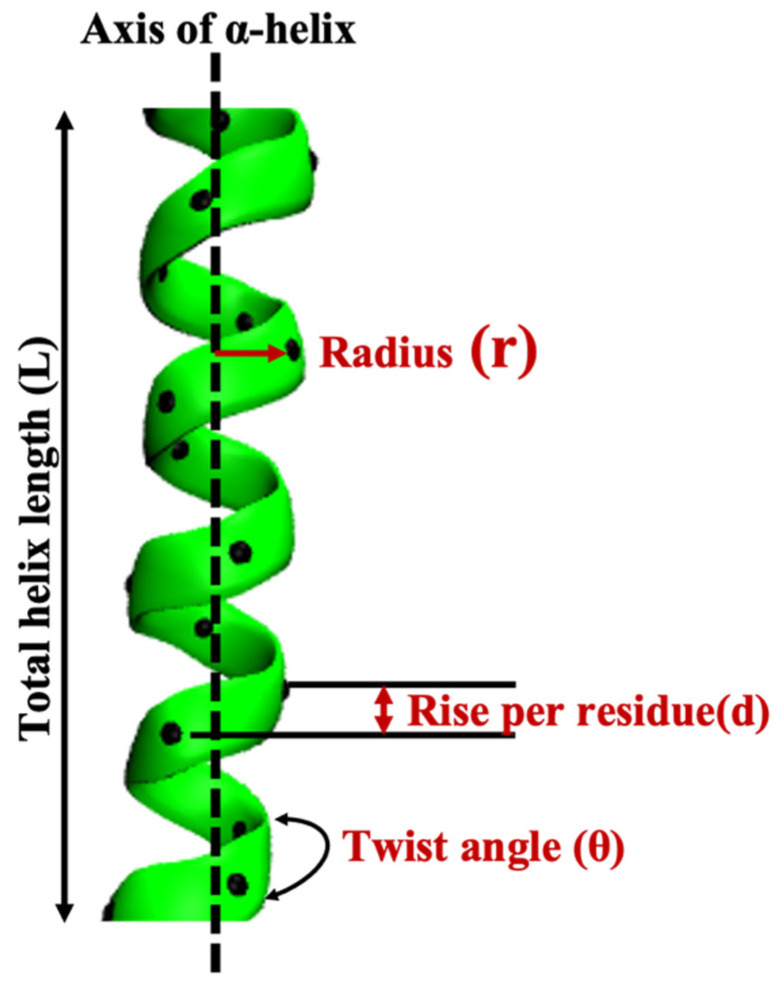
Schematic representation of helix properties.

Next, we investigate in detail the α-helical structures that both wtRop and RM6 attain at their native states, as well as their dependence on temperature. The conformation of an α-helix can be characterized via specific metrics, which are schematically presented in Figure 9 and are the following [54,55]: (i) Rise per residue, (d), is the distance in nm between sequential residues along the helix axis, which is calculated based on the positions of Ca atoms of the residues. (ii) Total helix length, (L), is the total length of α-helix in nm and it is defined as the product of the average rise (d) and the number of residues of the helix (n), as depicted in Figure 9. (iii) Helix radius, (r), which is defined as follows: $r=\sqrt{\frac{\sum_{i}^{N}(x{\left(i\right)}^{2}+y{\left(i\right)}^{2})}{N}}$, where *N* is the number of Ca atoms of the helix backbone. The helix radius is the radius of a circle centered on the axis of the helix with the Ca atoms on its periphery (Figure 9). (iv) Twist angle, (θ), is the average helical angle that is formed between two successive Ca atoms with the helix axis.

Averaging each property has been calculated along the α-helix at each time frame, and then block averaging was performed over the last 100 ns of the trajectories. The error bars were computed as standard deviation between the values of the blocks. Further averaging over the two/four α-helices of wtRop/RM6 was performed.

Average values of all the properties of α-helix for both proteins are shown in Table 5, where the second, third, fourth, and fifth columns depict the rise per residue, the total helix length, the helix radius, and the twist angle, respectively. Results indicate that temperature increasing affects some conformational characteristics of α-helices. More specifically: (a) T-increasing slightly affects the rise per residue (d) in both proteins at the highest temperature. (b) A small elongation of both proteins with T-rising is observed through L, which is also found in the end-to-end distance (Ree) calculations, as presented in Table S3 in the Supporting Material. Ree is defined as the distance between the first atom of the first residue and the last atom of the last residue that participates in the helical part for each subunit. This slight extension of α-helix is in agreement with the decrease in the number of the formed HBs within the α-helix region, discussed above. (c) A slight increase is observed in the helix radius with T-rising for wtRop, while there is no T-effect in RM6. (d) Finally, a decrease in the values of twist angle is observed by increasing the temperature for wtRop, whereas twist angle increases with temperature in RM6, which means correspondingly tighter and looser helices. The structural changes that temperature induces become clearer by monitoring the time evolution of the average value of the helix properties: d, L, r, and θ which are presented in Figures S9–S12, respectively, in Supporting Materials for both proteins. The deformation of wtRop is obvious through all measures with r and θ the most affected by temperature rising. At the same time the effect of temperature is considerably smaller on these two properties in RM6 which highlights its higher thermostability.

## 3. Discussion

The folding of wtRop protein is a widely studied topic providing valuable information in order to use it as a model protein. Moreover, its small size and simple and malleable structure establish wtRop protein as an attractive candidate for the development of novel biomaterials based on 4-α-helix bundles. Atomistic details are explored in order to analyze the key factors which affect the properties of the protein through the study of its RM6 loopless mutation. The focus is on the role of the loop region in the structure and the properties of Rop and RM6. Structural changes induced by temperature and the most sensitive parts of the structure to temperature changes are explored. The thermal stability of both proteins is investigated, highlighting the effect of temperature on their physical properties and, at the same time, indicating the differences between the two to temperature stimuli.

Atomistic MD simulations at three different temperatures: 300 K, 350 K, and 368 K were performed for both wtRop and RM6. Atomic resolution of our simulations high-lights more detailed features of temperature dependence, which can be crucial knowledge for the engineering of desired functionalities. In general, RM6 is indicated as more thermostable than wtRop through all studied measures. Root mean square deviation (*rmsd*) curves show that the effect of temperature on the structure of RM6 is observed at higher values (i.e., 368 K) compared to wtRop, where it is obvious from 350 K. Our findings are in good agreement with experimental data from circular dichroism regarding the melting temperature (Tm) [37], which indicates Tm ≥ 331 K for wtRop, while Tm ≥ 363 K for RM6. Pointing to more specific information and in agreement with *rmsd* results, root mean square fluctuations (*rmsf*) analysis highlights the higher thermostability of the RM6 mutant, also pointing to the more thermo-sensitive parts of both proteins. Deviations from native structures are detected mostly in the tail and loop regions. *rmsf* specifies that the loop region neighborhood (i.e., residues 25–33) of wtRop displays high flexibility at any temperature and is highly affected by temperature increase. However, the most flexible regions are the N-terminal (residues 1–3) and the C-terminal (residues 57–63) regions, in accordance with the crystallographic observations. The same observation stands for N-terminal and C-terminal residues of RM6 (i.e., 1–13 and 35–58, respectively).

Hydrogen bonding analysis shows a general trend of decreasing number of hydrogen bonds with increase of temperature in both proteins. Interesting is the loop region of wtRop where in its native state, 31Ala residue acts as a bridge, creating hydrogen bonds with both α-helices of a chain simultaneously. Temperature rising causes either the destruction of hydrogen bonds or the formation of new ones or both. Furthermore, loss of hydrophobic contacts at higher temperatures affects the extent of the hydrophobic core. This is reflected both in the interchain/interpair, (for wtRop/RM6, respectively), pair radial distribution functions between the C_β_ carbon atoms of the hydrophobic residues, as well as in the distance between the centers of mass of all the C_β_ carbon atoms, which belong to the hydrophobic residues, of each chain/pair of the two proteins.

The temperature dependence of the observed conformations is given through the analysis of Ramachandran plots as well as via specific metrics which characterize α-helical structures. The effect of temperature on the conformational properties of α-helices is as follows: (a) Small elongation of both proteins with T-rising is observed through L, which is in agreement with the decrease in the number of the formed HBs within the α-helix region. (b) A slight increase is observed in the helix radius with T-rising for wtRop but not for RM6. (c) Decrease in the values of twist angle is observed by increasing the temperature for wtRop, whereas twist angle increases with temperature in RM6, which means tighter and looser helices, respectively. (d) T-increasing slightly affects the rise per residue (d) in both proteins.

It is worth noting here that wtRop and RM6 exhibit different physical characteristics even in their native states. The main difference between their structures is the loop region. The interruption of the heptad repeat pattern in wtRop, which is uninterrupted in RM6, gives rise to conformational changes with the most important ones being the topology of the resulting helical bundles, affecting their sizes, with the tetrameric RM6 being twice as large as the dimeric wild-type protein. The absence of the flexible loop region in RM6, along with its bigger size, and its extended hydrophobic core, leads to different physicochemical properties and plays crucial role in protein stability as well. Fur-thermore, the larger number of hydrogen bonds in the RM6 tetramer enhances its stability compared to the wild-type protein. In addition, the intrachain hydrogen bonding per amino acid constitutes an energetic measure for comparison. Values are bigger in wtRop, which can possibly excuse its smaller helix radius, compared to RM6. Temperature rising does not alter the correlations of all characteristics between the two proteins.

## 4. Systems and Methods

### 4.1. Experiments

CD spectra were obtained using a J-810 CD spectropolarimeter (Jasco Inc., Easton, MD, USA). Thermal denaturation was analyzed by collecting full far-UV CD spectra (260–190 nm) in the three different temperatures and monitoring the change of the typical α-helical minima at 208 and 222 nm. For collection of spectra, a quartz cuvette of 1-mm path length was used and protein concentrations of 0.15 mg/mL in 20 mM Tris HCl buffer, pH 8.0, with 5 mM NaCl. Far-UV spectra (260–190 nm) were measured with 50 nm/min scanning speed, a 2-min response time, and three accumulations. The Spectra Manager program (Jasco) was used for buffer subtraction and unit conversions to mean residual ellipticities (MRE).

### 4.2. Simulations

Initial (native) structures were obtained from the Protein Data Bank (PDB): Nuclear magnetic resonance (NMR) structure of wtRop protein (PDB code: 1RPR [32]) and X-ray structure of RM6 (PDB code: 1QX8 [19]). For the RM6 protein, the complete tetrameric molecule was built from the atomic coordinates of the content of the crystallographic asymmetric unit (i.e., two antiparallel chains) via application of a crystallographic dyad using the PISA software [56]. Moreover, the crystal structure of the RM6 protein did not include the flexible N-(four residues) and C-(seven residues) termini of the four subunits, which were added to the model system using the PyMol software package [57]. For both proteins the hydrogen atoms were added through the GROMACS software tool [58,59].

**Figure 10:**
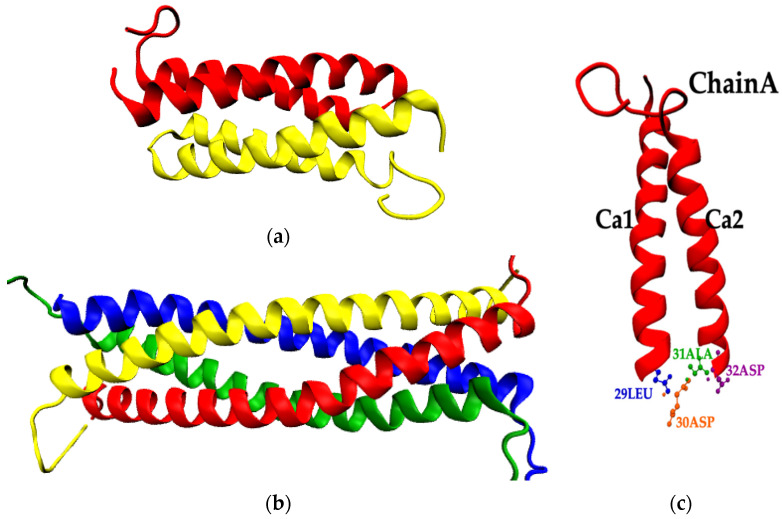
Graphical representation of (**a**) wtRop; (**b**) RM6 proteins—each color indicates a different subunit; (**c**) a subunit of wtRop (ChainA) distinct representation of the loop residues 29Leu (blue), 30Asp (orange), 31Ala (green), 32Asp (purple), and the two helices of a subunit.

All-atom molecular dynamics simulations using an explicit solvent model were performed with GROMACS software package [58,59]. Parameters for the description of intermolecular and intramolecular interactions for wtRop and RM6 proteins were taken from the GROMOS53a6 force field [60], while the SPC/E model was used to explicitly simulate water molecules [61]. The initial structures of proteins were solvated in a cubic simulation box and periodic boundary conditions (PBC) were applied in all three directions. In all cases, the simulation box was chosen to be ~1.5 times of the end-to-end distance (Ree) of the proteins. After the generation of the system energy minimization was applied using the steepest descent algorithm with tolerance of 1000 kJ mol^−1^ nm^−1^ and time step of 0.01 fs. The stochastic velocity rescaling thermostat [62] and the Berendsen barostat [63] were applied to maintain the temperature at the studied values (300 K, 350 K, and 368 K) and the pressure of 1 atm. The particle mesh Ewald (PME) summation was used for the computation of the electrostatic interactions, with a cutoff distance of 1.0 nm. The time step for the integration of the equations of motion was 1 fs, whereas the production runs were 270 ns and 200 ns for wtRop and RM6, respectively. A part of about 100 ns of the simulations were used for equilibration purposes, whereas the rest for the calculation of the static and dynamic properties of the systems. For both proteins, initial configurations were the same for all three temperatures and typical snapshots are illustrated in Figure 10a,b for wtRop and RM6, correspondingly. For clarity, solvent molecules are omitted.

## 5. Conclusions

A detailed investigation of structural conformational and physicochemical properties of wtRop and its RM6 mutation is presented in the current study. Atomistic MD simulations of model wtRop and RM6 were performed at three different temperatures: 300 K, 350 K, and 368 K. Simulation results, based on all studied measures, indicate that RM6 is more thermostable than wtRop. Moreover different physical characteristics of wtRop and RM6 proteins are found even in their native states. Experimental data from circular dichroism clearly demonstrate the effect of temperature on the integrity of the secondary structure of wtRop and RM6 (with the same temperature range used as in the simulation studies). In agreement with simulation results, experimental data show a change in the melting temperature of wtRop when it mutates to RM6 (moving to higher temperature values). The key difference between the structures of the two proteins is the loop region, where the interruption of the heptad repeat pattern in wtRop, cauces big conformational changes in the topolology and the size of the molecules.

The information deduced from the present work targets the atomic level, providing critical insights into the possibilities of small-scale changes in protein topologies. The observation of the mutations of wtRop reveals a great plasticity of this structural motif. This is used as a source of inspiration to design mutations of wtRop, such as its loopless mutation RM6 and to explore their potential use as building blocks to create new bioinspired materials. The study of the structural and physicochemical properties of the simulated systems in atomistic detail, summarized in the Section 3, helps in understanding the driving forces of the observed conformational changes. The detailed knowledge of the thermal response of proteins can be thought of as a guide for designing thermostable functional proteins for a variety of biotechnological applications. Further investigation of short-range changes (new mutations) concerning their physicochemical properties, as well as more collective properties, such as the propensity of creation supramolecular structures (e.g., fibrils), will constitute a future coordinated simulation and experimental study.

## Figures and Tables

**Table 1:** Details of the model systems: The number of protein molecules (Np), the number of solvent molecules (Ns), the total number of atoms in the system (N), the number of ions (Nions), the temperature (T), and the dimensions of the simulation box (L).

System	Np	Ns	N	Nions	T (K)	L (nm)
NSR1	1	24,189	73,861	14	300	9.0
NSR2	1	24,189	73,861	14	350	9.0
NSR3	1	24,189	73,861	14	368	9.0
MRM1	1	97,833	295,891	16	300	15.0
MRM2	1	97,833	295,891	16	350	15.0
MRM3	1	97,833	295,891	16	368	15.0

**Table 2:** Average number of hydrogen bonds between protein atoms, <P-P>, water molecules, <W-W> and protein-water molecules, and <P-W>for all model systems.

Systems	<P-P>	<P-W>	<W-W>/W
NSR1	125.2 ± 1.9	303.3 ± 2.9	3.6
NSR2	113.4 ± 1.7	293.8 ± 5.8	3.4
NSR3	113.1 ± 2.1	283.2 ± 2.9	3.3
MRM1	225.8 ± 3.5	517.4 ± 5.6	3.6
MRM2	221.1 ± 0.8	481.5 ± 3.2	3.4
MRM3	207.8 ± 2.4	482.1 ± 6.2	3.3

**Table 3:** Average number of hydrogen bonds between the *i*th and (*i* + 4)th residues of each α-helix separately of the systems.

Systems	HB_α-helix_ (Ca1/Ca)	HB_α-helix_ (Ca2/Cb)	HB_α-helix_ (Cb1/Cc)	HB_α-helix_ (Cb2/Cd)
NSR1	17.9 ± 0.13	15.9 ± 0.2	19.9 ± 0.1	19.7 ± 0.1
NSR2	15.8 ± 0.4	13.0 ± 0.2	14.7 ± 2.0	19.0 ± 0.1
NSR3	16.5 ± 0.9	15.2 ± 0.2	16.8 ± 0.3	14.2 ± 0.7
MRM1	37.0 ± 0.6	28.6 ± 0.4	34.9 ± 0.6	40.1 ± 0.7
MRM2	34.3 ± 1.6	36.7 ± 0.5	34.9 ± 0.4	37.7 ± 0.5
MRM3	29.8 ± 1.5	30.8 ± 1.5	33.3 ± 0.4	29.5 ± 0.1

**Table 4:** Average number of hydrogen bonds between every residue of the loop region and each individual α-helix for each chain.

<HBs>	NSR1	NSR2	NSR3
29Leu-Ca1	0.96 ± 0.01	0.9 ± 0.05	0.05 ± 0.04
29Leu-Ca2	0	0	0
29Leu-Cb1	0.97 ± 0.01	0.11 ± 0.02	0.9 ± 0.01
29Leu-Cb2	0	0	0
30Asp-Ca1	0.32 ± 0.11	0.5 ± 0.1	0.06 ± 0.04
30Asp-Ca2	0	0	0.3 ± 0.4
30Asp-Cb1	0.38 ± 0.2	0.05 ± 0.04	0.3 ± 0.35
30Asp-Cb2	0	0.01 ± 0.2	0
31Ala-Ca1	0.47 ± 0.17	0.55 ± 0.14	0
31Ala-Ca2	0.88 ± 0.07	0.72 ± 0.2	0.87 ± 0.13
31Ala-Cb1	0.47 ± 0.23	0	0.6 ± 0.03
31Ala-Cb2	0.92 ± 0.03	0.94 ± 0.03	0.77 ± 0.14
32Asp-Ca1	0	0	0
32Asp-Ca2	1.6 ± 0.04	1.51 ± 0.14	1.03 ± 0.14
32Asp-Cb1	0	0	0
32Asp-Cb2	1.54 ± 0.1	0.2 ± 0.1	1.4 ± 0.02

**Table 5:** Average values of the properties of an α-helix for all the studied systems.

Systems	d (nm)	L (nm)	r (nm)	θ (°)
NSR1	0.150 ± 0.005	3.46 ± 0.42	0.24 ± 0.01	99.09 ± 1.22
NSR2	0.153 ± 0.004	3.57 ± 0.53	0.26 ± 0.02	90.46 ± 8.64
NSR3	0.155 ± 0.005	3.63 ± 0.55	0.26 ± 0.02	90.01 ± 5.79
MRM1	0.146 ± 0.001	6.62 ± 0.21	0.33 ± 0.01	76.67 ± 5.92
MRM2	0.146 ± 0.001	6.64 ± 0.21	0.33 ± 0.01	78.16 ± 3.47
MRM3	0.150 ± 0.004	6.79 ± 0.21	0.33 ± 0.04	82.80 ± 4.62

## Data Availability

Statement is excluded.

## Supplementary Materials

Supplementary materials can be found at https://www.mdpi.com/article/10.3390/ijms22115931/s1.

## Abbreviations

MD	Molecular dynamics
CD	Circular dichroism spectroscopy
*rmsd*	Root mean square deviation
*rmsf*	Root mean square fluctuation
PDB	Protein data bank
NMR	Nuclear magnetic resonance
