# Supplementary material for: Structure and Thermal Stability of wtRop and RM6 Proteins through All-Atom Molecular Dynamics Simulations and Experiments
Source: Int J Mol Sci. 2021 May 31;22(11):5931. doi: 10.3390/ijms22115931 (PMC8199364; doi:10.3390/ijms22115931)
Supplement: Supplementary file 1 [file ijms-22-05931-s001.zip › ijms-1214376-supplementary.pdf]

International Journal of
Molecular Sciences

MDPI

Supplementary Materials

Structure and Thermal Stability of wtRop and RM6 Proteins through All-Atom Molecular Dynamics Simulations and Experiments

Maria Arnittali [1,2], Anastassia N. Rissanou [1,2,*], Maria Amprazi [3,4], Michael Kokkinidis [3,4] and Vagelis Harmandaris [1,2,5]

1 Institute of Applied and Computational Mathematics (IACM), Foundation for Research and Technology Hellas, (FORTH), IACM/FORTH, GR-71110 Heraklion, Crete, Greece.; maria.arnittali@iacm.forth.gr (M.A.); harman@uoc.gr(V.H.)
2 Department of Mathematics and Applied Mathematics, University of Crete, GR-71409, Heraklion, Crete, Greece
3 Department of Biology, University of Crete, GR-71409 Heraklion, Crete, Greece;
4 Institute of Molecular Biology and Biotechnology, Foundation of Research and Technology, GR-70013 Heraklion, Crete, Greece; amprazi@imbb.forth.gr (M.A); kokkinid@imbb.forth.gr (M.K.)
5 Computation-based Science and Technology Research Center, The Cyprus Institute, Nicosia 2121, Cyprus
* Correspondence: rissanou@iesl.forth.gr; Tel.: +30-2810-393746

Keywords: biomolecules; Rop; RM6; proteins; molecular dynamics simulations; mutations; thermostability; secondary structure

Sequences of wtRop and RM6 proteins

wtRop:

MT KQEKTAL NMARFIR SQTLTLL EKLNELD ADEQA DICESLH DHADELY RSCLARF GDDGENL

	f	g	a	b	c	d	e
**1**	(M	T)					
**3**	K	Q	E	K	T	A	L
**10**	N	M	A	R	F	I	R
**17**	S	Q	T	L	T	L	L
**24**	E	K	L	N	E	L	D
**31**	← **A** →			D	E	Q	A
**36**	D	I	C	E	S	L	H
**43**	D	H	A	D	E	L	Y
**50**	R	S	C	L	A	{R	F}
**57**	(G	D	D	G	E	N	L)

(a)

RM6:

MT KQEKTAL NMARFIR SQTLTLL EKLNEL - - - - -A DICESLH DHADELY RSCLARF GDDGENL

	f	g	a	b	c	d	e
**1**	(M	T)					
**3**	K	Q	E	K	T	A	L
**10**	N	M	A	R	F	I	R
**17**	S	Q	T	L	T	L	L
**24**	E	K	L	N	E	L	A
**31**	D	I	C	E	S	L	H
**38**	D	H	A	D	E	L	Y
**45**	R	S	C	L	A	{R	F}
**52**	(G	D	D	G	E	N	L)

(**b**)

Figure S1. Sequences of: (**a**) wtRop and (**b**) RM6 proteins with the assignment of heptad positions.

Root Mean Square Fluctuation

Figure S2 illustrates the rmsf values for the ChainA and ChainB of wtRop protein versus the residue index. The highest fluctuations are observed at 368 K, whereas the low values at 300 K indicate limited motion. The residues of the loop region (residues 28–32) seem to be more flexible at any temperature, especially at 368 K, where there is a jump in rmsf that becomes gradually lower at lower temperature. Moreover, the residues that move the most are the residues 1–3, which belong to N-terminal region and the last residues (residues 57–63) which belong to the tail and the C-terminal region.

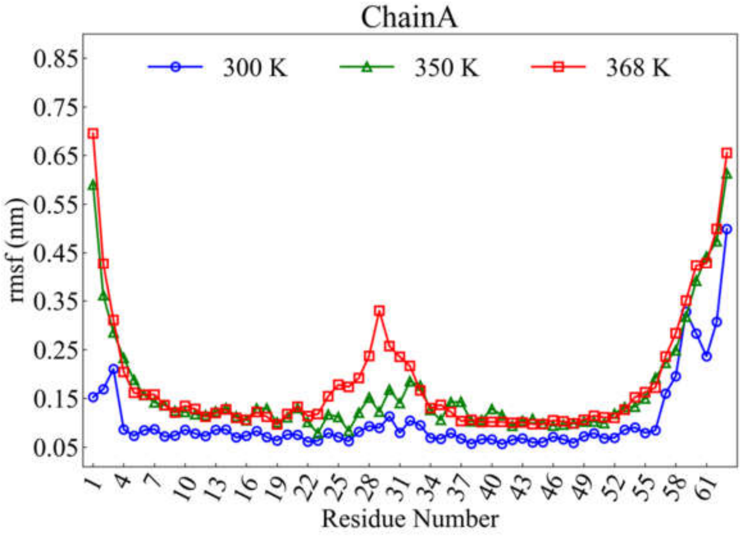

(**a**)

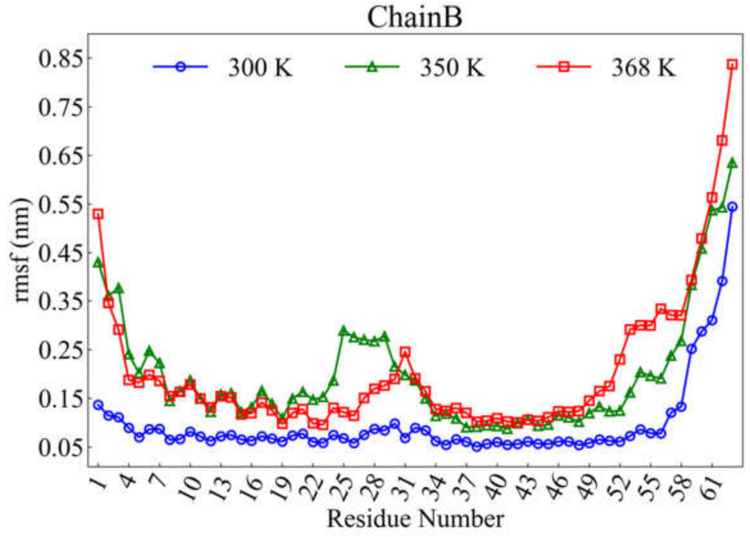

(**b**)

Figure S2. Average root mean square fluctuations (rmsf) of Ca atoms between the: (**a**) ChainA and (**b**) ChainB of wtRop protein per residue at 300 K (blue), 350 K (red), and 368 K (green).

Distance between the center of mass (CM) of interchain/interpairs

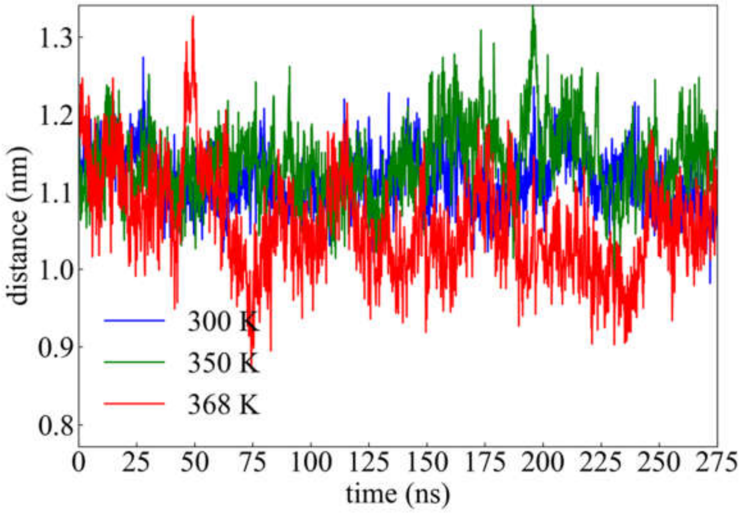

(**a**)

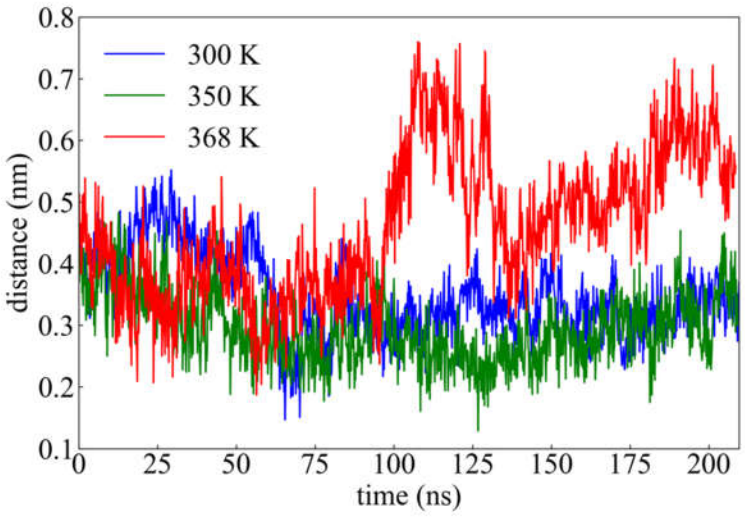

(**b**)

Figure S3. Distance between the centers of mass of all Cβ atoms that belong to the hydrophobic residues each chain/pair for: (a) wtRop protein; and (b) RM6 protein respectively, at the three different temperatures.

Hydrogen Bonds

A more comprehensive analysis of HBs is performed based on their division in interchain HBs (between different chains, i.e., HBs of ChainA-ChainB) and intrachain (within the same chain, i.e. HBs of ChainA-ChainA). In the following, the notation Ca, Cb, Cc, and Cd refer to ChainA, ChainB, ChainC, and ChainD, respectively.

Table S1 contains the average number of interchain hydrogen bonds among the various chain combinations. For wtRop an increase is observed at higher temperatures (350 K and 368 K) not in a monotonous way. In terms of RM6 protein, not any particular trend with temperature can be assigned. Differences are observed between the different pairs of chains (e.g., < Ca – Cb > and < Cc – Cd >). Even bigger differences are found between not equivalent pairs of chains. (e.g., for < Ca – Cb > and < Ca – Cd >), where Ca and Cb have an antiparallel orientation while Ca and Cd have a parallel orientation. Distances between these pairs are also different.

Table S1. Average number of interchain hydrogen bonds among the various combinations of chains.

System	<Ca-Cb>	<Ca-Cc>	<Ca-Cd>	<Cb-Cc>	<Cb-Cd>	<Cc-Cd>
NSR1	7.3±0.6	-	-	-	-	-
NSR2	8.5±1.2	-	-	-	-	-
NSR3	7.9±0.5	-	-	-	-	-
MRM1	8.8±1.4	5.7±0.4	1.5±0.5	0.7±0.6	4.9±1.3	11.1±1.8
MRM2	10.1±0.2	4.8±0.3	1.1±0.6	0.3±0.3	5.9±0.2	19.4±0.8
MRM3	12.2±3.5	9.7±1	3.4±2.7	0.1±0.1	4±0.8	10.4±1.3

Table S2 contains the average number of interchain hydrogen bonds among the various pairs of chains. In all cases a reduction of the intrachain hydrogen bonds is noticed with temperature increasing which can be attributed to the conformational changes of α-helices.

Table S2. Average number of intrachain hydrogen bonds among the various combinations of chains.

System	<Ca-Ca>	<Cb-Cb>	<Cc-Cc>	<Cd-Cd>
NSR1	60.2±1.23	58.6±0.2	-	-

NSR2	54.7 ±1.7	49.9±0.8	-	-
NSR3	52.3±1.3	53.1±0.6	-	-
MRM1	46.7±0.8	50.5±0.4	43.6±1.6	52.5±1
MRM2	44.7±0.4	48.7±1.1	40.9±0.5	45.4±0.5
MRM3	40.6±1.4	40.5±1.4	41.1±0.2	45.9±0.8

Hydrogen bonds (HB) play an important role in the stability of the secondary structures, such as α-helix and β-sheet, in proteins. Figure S4 represents the formation of a hydrogen bond between the ith and (i+4)th hydrogen bonds of an α -helix.

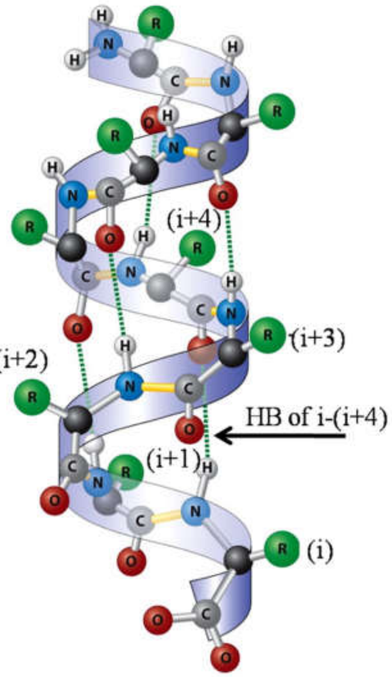

Figure S4: The hydrogen bond pattern formation of an α-helix between ith and (i+4)th residues.

Ramachandran Plot

Figure S5 contains the Ramachandran plot of wtRop at the various temperatures: at 300 K (S5a), at 350 K (S5b), and at 368 K (S5c).

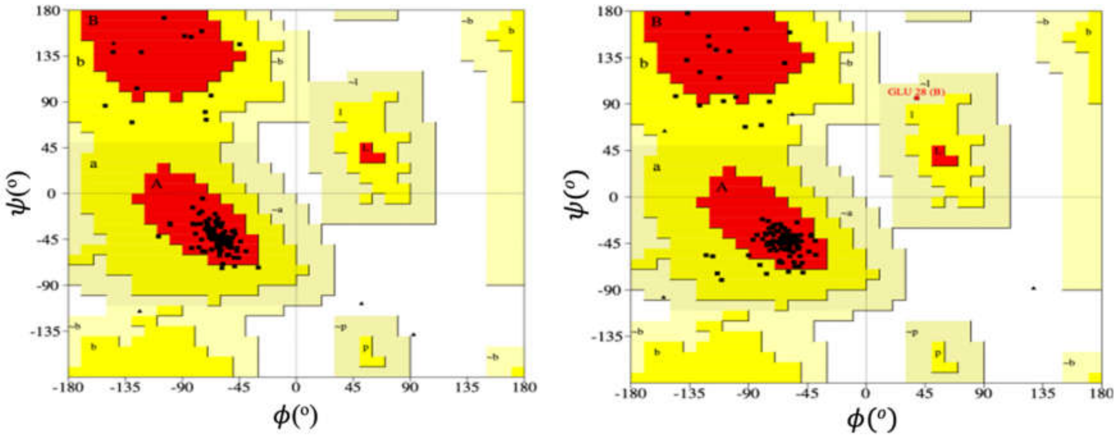

(**a**) (**b**)

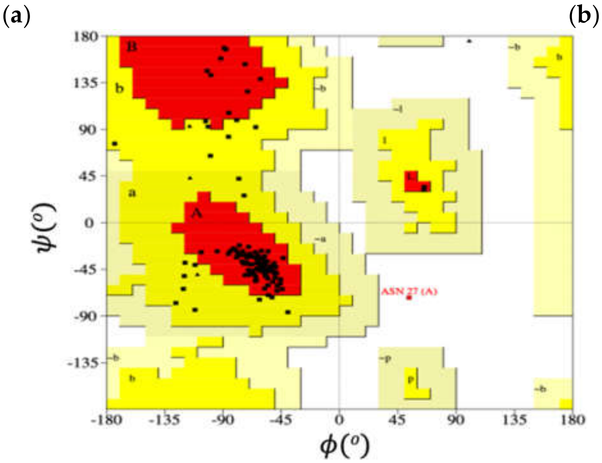

(c)

Figure S5. Ramachandran plot of wtRop protein at three different temperatures (a) 300 K, (b) 350 K, and (c) 368 K. Colours indicate how favourable is an area for a combination of (φ,ψ) angles. The red colour shows the most favourable region, whereas the gradient of yellow colour from the darker to brighter regions shows the passage to the less favourable conformations.

Map of Dihedral Angles of the loop residues:

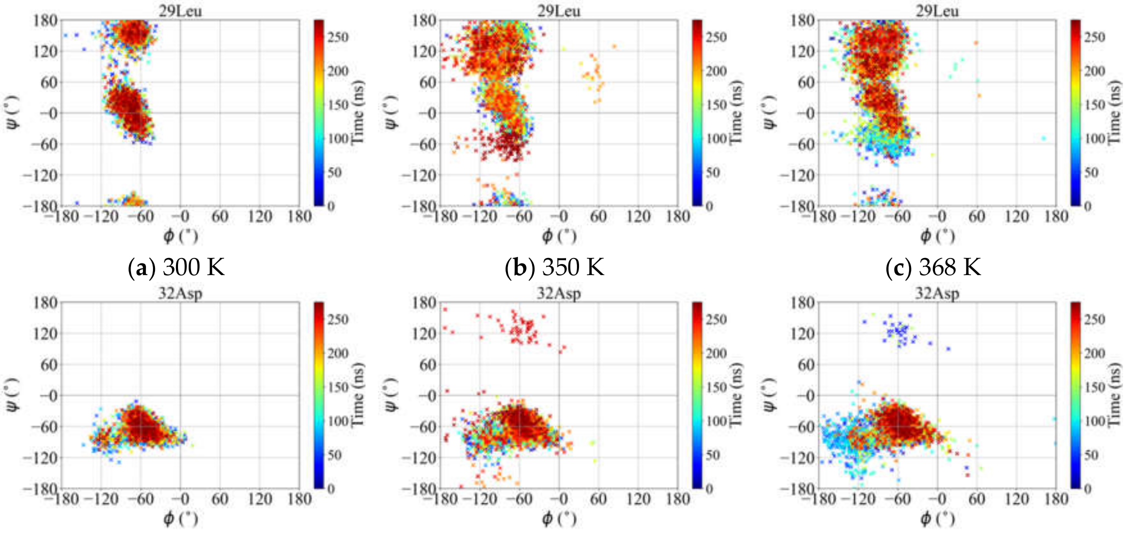

(**a**) 300 K (**b**) 350 K (**c**) 368 K

(**d**) 300 K (**e**) 350 K (**f**) 368 K

Figure S6. Map plots of all the (φ,ψ) torsion angles during the MD simulation. Colour code denotes the time instant in which the combination of (φ,ψ) is obtained. (a,b,c) 29Leu and (d,e,f) 32Asp.

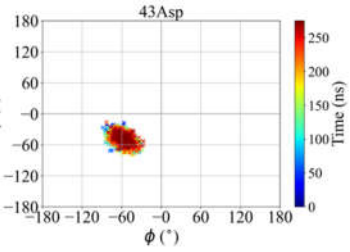

(**a**) 300K

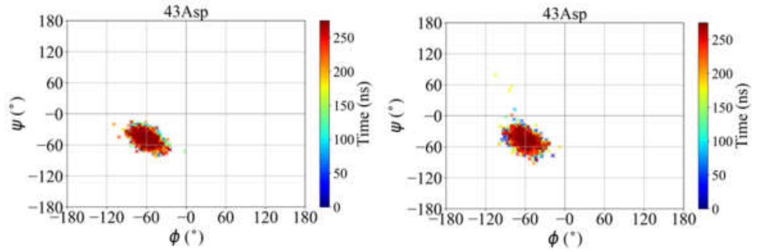

(**b**) 350K (**c**) 368K

Figure S7. Map plots of all the (φ,ψ) torsion angles for 43Asp during the MD simulation. Colour code denotes the time instant in which the combination of (φ,ψ) is obtained.

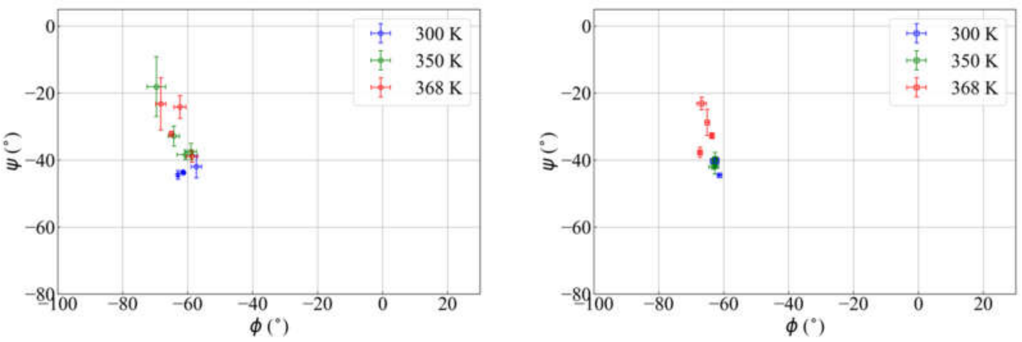

(**a**) (**b**)

Figure S8. Average dihedral angles of each α-helix per subunit of (a) wtRop protein (Figure 10c), and (b) RM6 protein (Figure 10b), at the three different temperatures.

Local Conformation Analysis of Alpha-Helices: Helix Properties

End to end distance (Ree): is the distance along the axis of the helix from the one end to the other. This is determined as the distance between the first atom of the first residue and the last atom of the last residue that participate in the helical part for each subunit. Averaging has been performed among the four α-helices at each time frame and then block time averaging is made over the last 100 ns of the trajectories. The error bars were calculated as the standard deviation between the values of the averages of the blocks. Table S3 contains the average values of the end-to-end distances for all the studied systems. An increase of the end-to-end distance with temperature is observed for wtRop, which points towards an extension of α-helices. In the case of RM6 protein, temperature effect is not detected.

Table S3. Average end to end distance (Ree) for all the studied systems.

System	R_{ee}(nm)
NSR1	3.5±0.34
NSR2	3.8±0.12
NSR3	3.6±0.47
MRM1	6.8±0.24
MRM2	6.8±0.22
MRM3	6.9±0.21

Figure S9 illustrates the time evolution of the average values of rise per residue of wtRop (a), and RM6 (b) at all three temperatures. In wtRop, the values tend to be stable around the value 0.15 nm at 300 K, whereas with the T-increasing a slight increase is observed after ~175 ns. In the case of RM6 protein, a slight increase of the d values is shown at 368 K, while at 350 K seems to be unaffected.

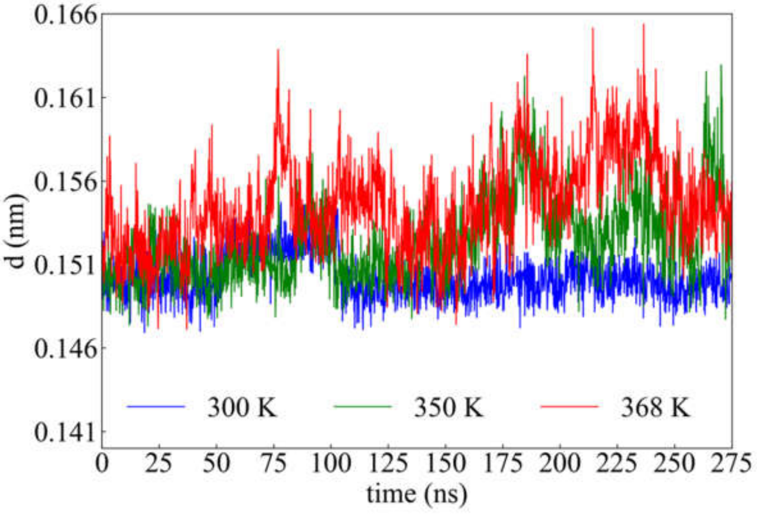

(**a**)

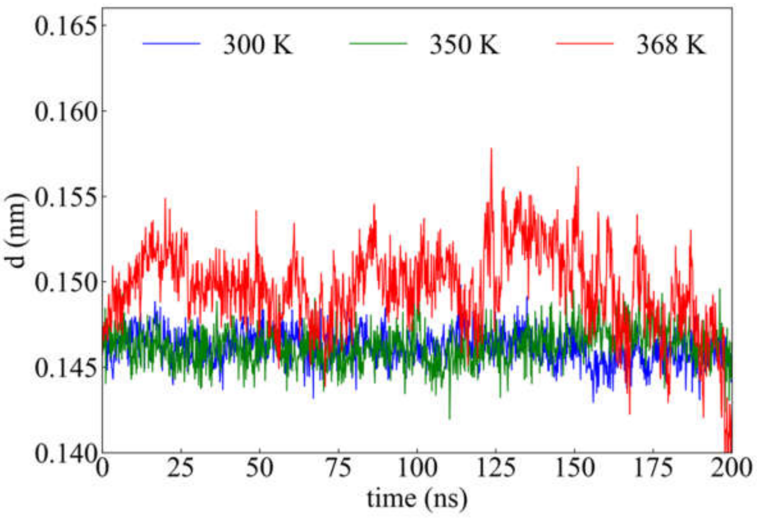

(**b**)

Figure S9. Time evolution of the average rise (d) of the α-helices of: (a) wtRop; (b) RM6, at 300 K (blue), 350 K (green) and 368 K (red).

Figure S10 shows the time evolution of the average values of the length of α-helices of wtRop (a), and RM6 (b) at all three temperatures. For wtRop at 300 K, the values tend to be stable around the value 3.46, whereas T-increasing leads to a small increase to values ~(3.57 - 3.63) after ~175 ns. In RM6 protein, the length seems to be unaffected at 350 K, whereas a small increase is observed at 368 K.

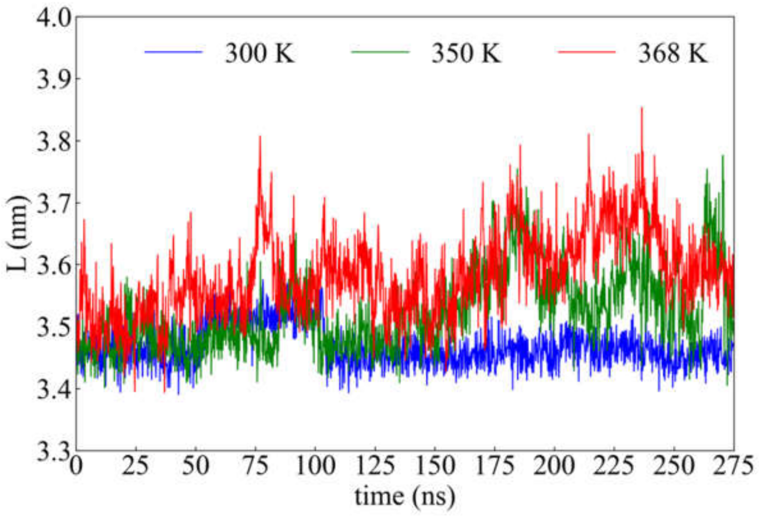

(**a**)

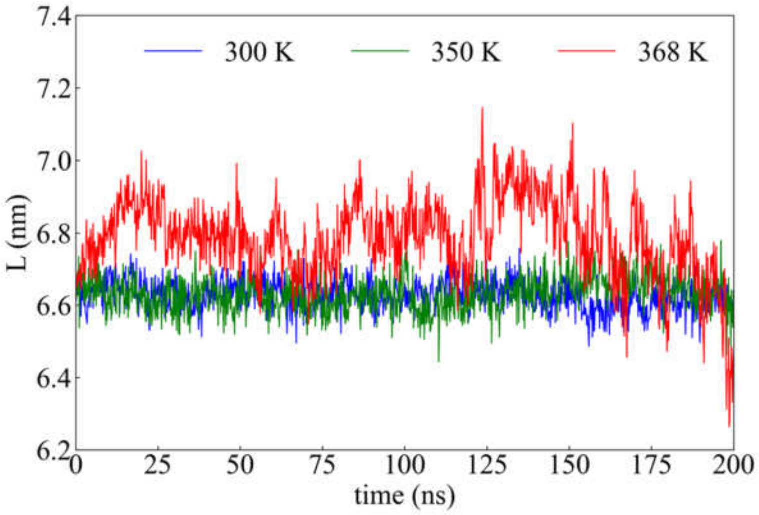

(**b**)

Figure S10. Time evolution of the average length (L) of the α-helices of: (a) wtRop; (b) RM6, at 300 K (blue), 350 K (green) and 368 K (red).

In Figure S11 the time evolution of the average radius of the α-helices of wtRop (a), and RM6 (b) for all systems is presented. In wtRop, the values tend to be almost stable at 300 K, whereas T-rising lead to increase of r beyond ~175 ns. On the contrary the increase of temperature does not affect the radius of α-helices in RM6 protein.

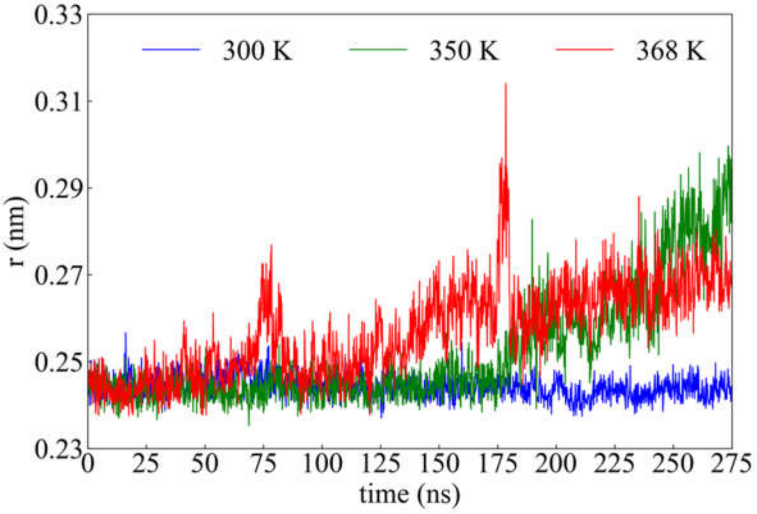

(**a**)

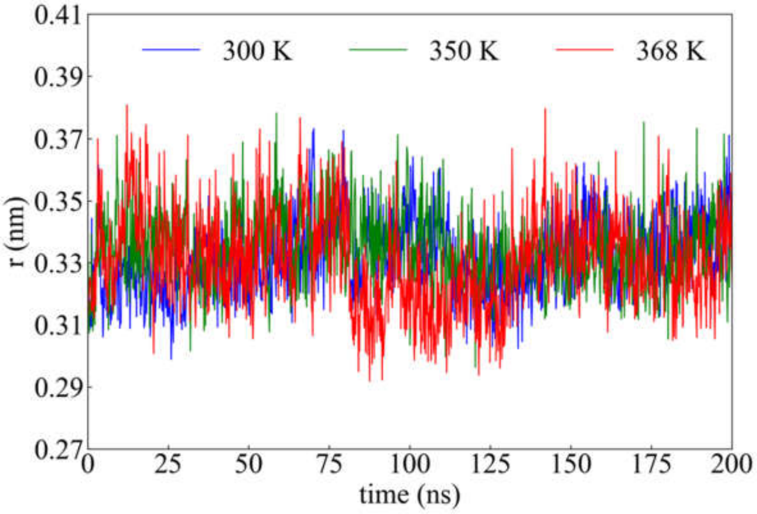

(**b**)

Figure S11. Time evolution of the average radius (r) of the α-helices of: (a) wtRop; (b) RM6, at 300 K (blue), 350 K (green) and 368 K (red).

In Figure S12 the time evolution of the average twist angle of the α-helices of wtRop (a), and RM6 (b) is shown for all systems. In wtRop, the values attain stable values at 300 K, whereas with the temperature rising induces a considerable decrease of its value beyond ~100 ns. On the other hand increase of T has negligible effect in the twist angles for RM6 protein.

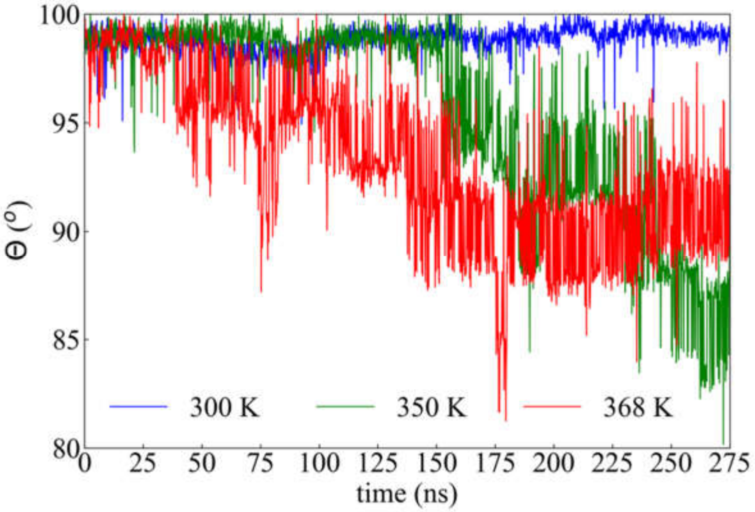

(**a**)

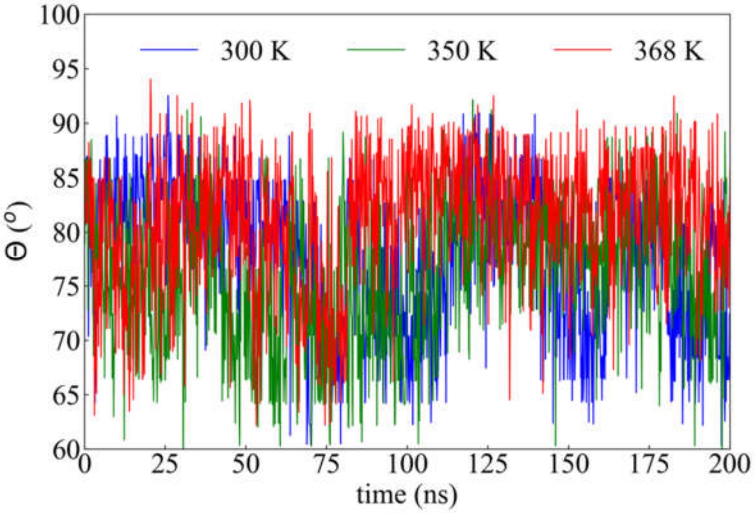

(**b**)

Figure S12. Time evolution of the average twist angle (θ) of the α-helices of: (a) wtRop; (b) RM6, at 300 K (blue), 350 K (green) and 368 K (red).

© 2021 by the authors. Licensee MDPI, Basel, Switzerland. This article is an open access article distributed under the terms and conditions of the Creative Commons Attribution (CC BY) license (http://creativecommons.org/licenses/by/4.0/).
